# Targeted genome and epigenome editing using engineered TALE and CRISPR/Cas9 technologies

**DOI:** 10.1186/1744-8069-10-S1-O4

**Published:** 2014-12-15

**Authors:** Charles A Gersbach

**Affiliations:** 1 Department of Biomedical Engineering, Duke University, Durham, NC 27708, USA; 2 Center for Genomic and Computational Biology, Duke University, Durham, NC 27708, USA; 3 Department of Orthopaedic Surgery, Duke University Medical Center, Durham, NC 27710, USA

## 

The impact of reverse genetics, synthetic biology, and gene therapy has been restricted by the limitations of conventional genetic engineering technologies. To expand the capacity for genetic modification of mammalian cells, we are engineering artificial DNA-binding proteins, including zinc finger proteins, TAL effectors, and CRISPR/Cas9 to regulate and edit endogenous mammalian genes. For example, we have engineered both protein-based and RNA-guided transcriptional activators and repressors targeted to human genes relevant to medicine, science, and biotechnology. Delivery of combinations of transcription factors led to synergistic effects on gene activation and tunable expression levels. This approach recapitulates the previously intractable complexity of natural regulation of mammalian genes that is the product of cooperative actions of many transcription factors. We have also developed novel methods for controlling the activity of these proteins, such as optogenetic regulation of protein dimerization with blue light. Genome-wide analysis of the DNA-binding, gene regulation, and chromatin remodeling of these targeted epigenome modifiers has demonstrated their exceptional specificity. In other studies we have engineered synthetic nucleases to stimulate gene targeting to genomic safe harbor sites. This approach is particularly useful for generating isogenic cell lines. We showed that this method leads to a decrease in the variability of transgene expression within a clonal cell line and between multiple clones relative to conventional techniques. Finally, we have used similar methods to correct mutations causing genetic disease. We engineered synthetic nucleases targeted to the human dystrophin gene that is mutated in Duchenne muscular dystrophy patients. When we delivered these nucleases to cells from patients with this disease, the correct gene reading frame and expression of the functional dystrophin protein were restored *in vitro* and following cell transplantation *in vivo*. We further demonstrated that these nucleases were well-tolerated and did not lead to off-target alterations of the exome in several corrected clonal cell populations. Collectively, these studies demonstrate the potential of engineered DNA-binding proteins to enable new approaches in medicine, science, and technology.

## Disclosures

Charles Gersbach is an inventor on patent applications and consultant in the area of genome editing.

